# How political philosophies can help to discuss and differentiate theories in community ecology

**DOI:** 10.1007/s40656-021-00401-x

**Published:** 2021-04-09

**Authors:** Annette Voigt

**Affiliations:** Group of Open Space Development, Department of Architecture, Urban and Landscape Planning, University of Kassel, Kassel, Germany

**Keywords:** History and philosophy of ecology, Community ecology, Political philosophy, Analogy, Constitution theory

## Abstract

This paper uses structural analogies to competing political philosophies of human society as a heuristic tool to differentiate between ecological theories and to bring out new aspects of apparently well-known classics of ecological scholarship. These two different areas of knowledge have in common that their objects are ‘societies’, i.e. units composed of individuals, and that contradictory and competing theories about these supra-individual units exist. The benefit of discussing ecological theories in terms of their analogies to political philosophies, in this case liberalism, democratism and conservatism, consists in the fact that political philosophies show clear differences and particularities as regards their approach to the concepts of individuality and intentional action. The method therefore helps to expose peculiarities of ecological theories that are usually considered canonical (e.g. Clements, Gleason), as well as hybrid forms (E. P. Odum), and to differentiate between two different types of theories about functional wholes. The basis of this method is the constitutional-theoretical premise that modern paradigms of socialization structure the ecological discourse.

## Introduction

In the philosophy of ecology, theories of community ecology are discussed mainly against the background of the holism–reductionism debate. In the broadest sense, this debate is about the relationship between wholes and their parts or elements. In community ecology, the wholes under consideration are generally groups of organisms of different species (e.g., associations, communities, or ecosystems) and the parts are individual organisms (or groups of organisms): to be precise, therefore, what is at issue is the debate between individualism and organicism. Questions of whether, how, and to what extent individuals are integrated into groups represent the principal sources of disagreement. In studies of the history and philosophy of ecology, various approaches have been developed to discuss positions in the individualism–organicism debate and to classify and locate particular theories within this spectrum.^1^

My paper introduces a specific approach to the discussion of synecological theories, highlighting their various commonalities, differences and peculiarities. I use *analogies between ecological theories and political philosophies* for this discussion. These two different areas of knowledge have in common that their objects are ‘societies’, i.e. units composed of individuals. In both areas, there are different and contradictory competing philosophies or theories about the nature and development of the supra-individual units involved. But in comparison with ecological theories, in political philosophies certain aspects have been more clearly or distinctively elaborated and reinforced against attacks by their opponents. I use these differences, in particular those regarding *purposes* and the essential characteristics of the *individual*, as a basis for the discussion of ecological theories. The method I present here is to lay out three ideal–typical conceptions of individuality and society and to examine real ecological theories to see whether, and how far, they correspond to these conceptions. Thus, I show the structural analogies between political philosophies and synecological theories in respect of their assumptions about the organization and development of the biological or human supra-individual entity, as well as pointing out where the discrepancies between these two domains lie. I use these analogies as a heuristic tool to uncover new aspects of apparently well-known ecological classics. Knowledge of political philosophies helps to change our perspective and to ask further questions about ecological theories, because political philosophies and the associated secondary literature explicitly emphasize and discuss the points that I am interested in.

In the following, I undermine the separation between these fields of knowledge. I do not separate them because of their different subject matter, i.e. ‘human society’ and ‘synecological units’, instead I associate them because of their similarities. My intention is by no means to negate the difference between political philosophies and scientific theories or the difference between social and natural systems.^2^ My work is interdisciplinary—it connects two disparate fields of study and knowledge by pointing out structural analogies between political philosophies and ecological theories of socialization. Nevertheless, the reasons for the existence of these analogies are not located in ‘nature’. Instead, I present a theory of cultural constitution that can explain both why there are contradictory theories in synecology in the first place and why analogies exist between political philosophies and synecological theories.

Other authors, predominantly from Germany, have already laid out more or less extensively the structural analogies between conservative social theories and organicist ecological theories, as well as liberal theories and individualistic theories (see for example Eisel, 2002, 2004; Trepl, 1994a, b; Trepl & Voigt, 2011).^3^ In extension, I open up a third area of analogy, by drawing parallels between the democratic philosophy of the French Enlightenment and the ecosystem approach.^4^

## Preliminary methodological remarks

### Ideal types

How to present these analogies in a purposeful and practicable way? An approach that is appropriate for my concerns here is to consider political philosophies as *ideal types*, rather than referring to the philosophies of particular individuals as such. Following German sociologist Max Weber (1864–1920), an ideal type does not claim to correspond fully to a real position, but is a *heuristic* instrument for analytically grasping reality by comparison (Weber, 1904: 190, 203). It is important to note that in using the term *ideal*, Weber refers to the world of ideas and not to ‘ideal’ in the sense of perfect or optimal. For him, the aim of forming an ideal type lies primarily in heuristics, i.e. gaining new knowledge, and also in the training of judgment and in facilitating the formulation of new research hypotheses (ibid.: 190 ff.). Ideal types are useful tools for analysis and comparison, enabling us to identify the peculiarities of specific theories as well as the similarities between them. An ideal type owes its existence to the “one-sided accentuation of one or more points of view and by the synthesis of a great many diffuse, discrete, more or less present and occasionally absent concrete individual phenomena, which are arranged according to those one-sidedly emphasized viewpoints into a unified analytical construct” (ibid.: 191, translated). Therefore, an ideal type is a methodically guided exaggeration that does not exist in its ‘pure form’ in reality, but nonetheless represents a useful tool.

Thus, I will contrast the ideal types of liberal, conservative and democratic philosophy with certain synecological theories and point out their structural similarities. In doing so, I emphasize certain aspects of the ideal–typical constructions, namely those which make it possible to show analogies. These points of view concern the following questions: (a) What are the causes and factors of the coexistence of individuals or species? What is the nature of the *relationships* between the individuals as well as between the individuals and the whole? Does the social whole have a *purpose*, and is the individual subject to purposes in his individual and/or social actions? (b) How does the process of *development* take place? (c) What assumptions do these theories make about the state of reality of the whole, i.e. its *ontological status*?

I explain these ideal types by referring to historical examples, i.e. early political philosophies published by various German- and English-speaking authors. Following Weber’s view of an ideal type as a useful tool constructed by accentuation and omission, I do not discuss at first whether there are aspects of these examples that might speak for a different interpretation, but I highlight the aspects that help me to produce analogies and highlight contrasting positions. Later, I discuss some ways in which ecological theories diverge from these analogies.

### Early political philosophies and ecological theories

I take as my starting point the competing political philosophies of liberalism, democratism, and conservatism. Of course, in the broad spectrum of political philosophies of modernity there are also others such as anarchism or fascism, and furthermore there are all kinds of transitions and intermediate forms, but I will not go into these. Why have I chosen just these three political philosophies as starting points? I have two content-related reasons for choosing these theories: The selection results from the thesis that all modern political philosophies are not only political concepts of the ‘reasonable’ government of the people by the people, but also take a particular stand on the fact and/or the idea of *progress* as well as on conceptions of *individuality*. The two extreme positions are one that affirms progress and one that rejects it, and these can be found respectively in classical liberalism and classical conservatism in the form in which they emerged in the seventeenth and eighteenth centuries. In response to the inadequacies inherent in these two political philosophies, diverse “third ways” (Gallus & Jesse, 2001; Sturm, 2001) have emerged that integrate theoretical elements of both in specific ways. Democratism (with socialism in its wake) is probably the most important third way, and was and is a historically powerful worldview. In addition, we only inadequately grasp the progressive position if we try to understand its thinking solely on the basis of liberalism. Even though we often do not distinguish between the notions of ‘liberal’ and ‘democratic’ in the present day, there are decisive differences at the level of political philosophy. Liberalism emerged in the Anglo-Saxon world as a philosophy and form of society that met the needs of a capitalist market society characterized by competition and exploitation. Within the framework of the liberal conception of society and individuality, the competition of *diverse* and *selfish interests* is what determines the nature of society. In contrast, the democratic philosophy that developed primarily in the course of the French Revolution does not aim to maintain the diversity of interests, but realizes the *general will*, i.e. the reasonable will of all citizens, which is directed impartially towards the interests of the community (cf. Nonnenmacher, 1989: 248 ff.). This also means that the three philosophies selected here developed different conceptions of individuality and society in a particularly consistent way: Do individuals act independently of a superior whole (liberalism)? Are they integrated into a superordinate whole, either in an individual way (conservatism) or in a general way (democratism)? Moreover, in choosing to treat these three philosophies, I am following a polarizing division often used in studies of the history of political ideas (compare e.g. Heidenreich, 2002; similarly Schoeps, 1981; Alexander, 2014 distinguishing between liberalism, conservatism and socialism).

While it might be exciting to discuss ecological theories against the backdrop of the diversity of current political positions and thus possibly identify parallels in contemporary thinking about community and development, the aim and method of this article is a different one. For the purpose of constructing ideal types of liberalism, democratism and conservatism, I am referring to political utopias that essentially emerged in Europe in the seventeenth and eighteenth centuries in response to fundamental social and economic changes at the beginning of modernity. The advantage of referring to earlier utopias lies in the fact that the *structural cores* are particularly prominent in these formulations and are therefore easier to identify and sharpen into ideal types than in later variants. After all, early formulations were not yet forced to take into account the diversity and contradictoriness of the modern world—or at least only to a lesser extent than later ones. For example, in contrast to the corresponding positions today, neither early liberalism nor early conservatism was democratic. Moreover, the structural core is specific to the respective political worldview; it marks the relation of certain statements and values, which remains constant in principle, even in the many variants of each philosophy and across the changes and political compromises to which it is subject. It is their shared liberal core, for example, that connects the philosophies of John Locke and Adam Smith with each other as well as with current liberal theories, despite all the differences between them. Thus, the construction of three ideal types of early political philosophies as opposing extremes is useful for the precise comprehension of all political positions as well as for the discussion of ecological theories dating from the twentieth century. This is because the ‘ideal poles’ are a prerequisite for being able to identify the deviating empirical cases that make up the spectrum of reality as variants, mixtures, intermediate positions or real alternatives.

The reference to individual authors serves on the one hand to illustrate each ideal type. On the other hand, the texts of the authors are, in Max Weber’s sense, the material from which the ideal types are developed and at the same time the measure against which the suitability of the types can be tested (Weber, 1904: 193, 204 f.). I have chosen works for the formation and illustration of the ideal types that are canonical classics and have been subject to detailed discussion and debate. Crucially for me, the structural core of each political philosophy is made very clear in these works.

The selection of ecological theories follows similar considerations. I have selected ‘classic works’ that have made significant contributions to ecology and have influenced the discipline and its discourse then and now. In addition, I have selected theories whose structural core is relatively easily identifiable. In the history of theories of ecology, the competing positions have each felt compelled to consider the empirical facts that supported the arguments of the opposing side. In debate, the presentation of facts rarely refutes positions completely; rather, the facts lead to the differentiation and refinement of the respective positions. If the ecological positions have also retained their structural core in the process, however, it becomes more difficult to identify—as in the case of political philosophies—because of adaptations, divergences, transformations and even syntheses. Thus, when I point to analogies between ecological theories and ideal–typical political philosophies in what follows, I refer primarily to older publications in the discipline of ecology, since the analogies are particularly clear here. In addition, however, I will give some hints as to where or to what extent we can also identify correspondences with more recent ecological theories.

## Analogies between the classical positions in ecology and political philosophies

### Analogies between liberal philosophies and synecological theories: ‘societies of independent individuals’

The ideal–typical construction of liberalism can be based on different variants of liberal theories that diverge with regard to the answers they give to the following questions: What characteristics do people have by nature? What kind of government is therefore necessary? Are society and/or the state the result of a contract between autonomous rational individuals or of a quasi-natural ‘rational’ evolution? However, they agree in affirming the empiricist denial of the existence of higher principles (or at least the possibility of knowing them). Humans are autonomous, i.e., everybody has an original right to the free disposal of his own person, and tries to assert his interests. There are different kinds of relations between people—mainly competition, but also cooperation within the framework of the division of labour. I refer to John Locke’s (1632–1704) and Adam Smith’s (1723–1790) political philosophies, which clearly show the same structural core but are otherwise quite different. Both are much-discussed classics among liberal empirical philosophies as they emerged in the course of the Enlightenment, especially in England in the seventeenth and early eighteenth centuries. Both formulate a view of society based on the concept of social progress and of the individual as an autonomous subject and therefore allow a clear demarcation from conservatism and from democratism. Smith’s variant of liberalism also reveals another aspect that is important for the ideal–typical construction of the ‘society of independent individuals’: The competition of the individual’s interests leads ‘naturally’ to social and political progress in capitalist society. Concerning ecology, probably independently of each other, quite different early ecologists developed theories which understood supra-individual units in an analogous manner; e.g. American botanist Henry A. Gleason (1882–1975), Russian ecologist Leonty G. Ramensky (1884–1953), and German entomologist Fritz Peus (1904–1978) (cf. Trepl, 1987: 236; Jax, 2002). I refer to these older theories because they allow the basic idea to be presented very clearly. However, analogies to the ‘society of independent individuals’ are also recognizable in later theories (see below).

(a) What are the causes and factors of the coexistence of individuals or species? What is the nature of the relationships between individuals, as well as between the individuals and the whole? Following the ideal type of liberalism, people are *autonomous*, i.e. they have an original right of free disposal over themselves and are not bound to a particular place in their society (Locke, 1690: 9). In society, they try to enforce their interests and are free to act within legislation that applies *equally* to all in order to satisfy their particular and selfish needs and accumulate wealth. There are different types of relationships between people—mainly competition, but also cooperation within the context of the division of labour.

Of course, in the corresponding ecological theories, ‘being independent’ does not mean freedom or autonomy. “[E]very species of plant is a law unto itself” (Gleason, 1939: 107 f.). Whether a species is or is not found in a particular place depends on internal factors (i.e., the organism’s properties and environmental requirements) and external factors (i.e., environmental factors, including the effects of other organisms), but a species does *not* require a particular society or combination of species. “The location and behavior of every individual plant is determined partly by heritable requirements and partly by the environmental complex under which it grows” (Gleason, 1927: 321). Furthermore, the self-production of organisms does not perform functions for a superior whole, i.e. the community. One organism may well serve an essential function for another (such as prey for a predator), but this does not explain its properties. According to interactionist ecologists, such as Ramensky (1926), *interspecific relationships*, especially competition and predation, decisively determine which species coexist.

In liberalism as well as in the corresponding type of ecological theory, the essential characteristics of individuals are in principle independent of other individuals and of the whole of society. The formation of society comes as a result of individuals’ interactions and their competition for scarce resources. Competitors develop certain behaviours to avoid competition, most notably specialization (or technical/behavioural improvements). Individuals also cooperate and thus mitigate competition or gain advantages over others (Nonnenmacher, 1989: 90 f.). In liberalism, the state has the purpose of securing and optimizing these interactions between individuals. Through socialization—in the form of interactions or a social contract—the individual gains advantages for him/herself. Society or socialization is beneficial or harmful solely from the perspective of the individual.

(b) The development of the liberal society—as well as that of each individual—has *no fixed endpoint*; it is in principle unfinished and open-ended. However, it can be said that the goal of progress is the emancipation of the citizens, i.e. detachment from their initial conditions and constraints. According to Adam Smith (1776), *free competition* in the market is the engine of progress for society and innovation. Within the market various niches differentiate themselves, leading to specialization and differentiation in both resource use and production and thereby allowing competition to be avoided. A division of labour emerges, which enables more effective production. Even if the intentions of the individuals are particular and egoistic, and their actions are not intentionally directed towards society, the economic common good of all is nevertheless and precisely because of their interplay. Besides, freedom of opinion and pluralistic dissent are also necessary for social progress.

Individualistic ecological theories express development in a similar way. Even if the species composition of a community does not change significantly over a long period, new competitors or predators could appear at any time due to migration, evolution or behavioural change. Additionally, the abiotic conditions can change in such a way that the interspecific network of relationships also changes. “Stability of all vegetational causes never exists: physiographic changes never cease; climatic changes are probably always in operation; evolution of species is probably as rapid now as in any past period in the history of vegetation; migration of plants is constant” (Gleason, 1927: 322). Development is open and ‘unfinished’, and not, as in organicism, a goal-oriented development toward a stable climax.

(c) According to John Locke’s philosophy (1690), society is created by interactions between individuals. Therefore, in principle, individuals can change ‘their’ society, depending on who they interact with. The state and its legislation become necessary to secure, optimize and institutionalize the interactions of mutual benefit as well as the natural rights to freedom and property that already exist in the natural state of society, but have come into crisis (Nonnenmacher, 1989: 102). The state is an institution constructed by the citizens, to which autonomous subjects subordinate themselves voluntarily and out of benefit-oriented interest.

There are different positions about the ontological status of the synecological unit in individualistic ecology. Concerning interactionistic theories, which assign a significant impact to *interspecific relationships*, synecological units can be interpreted in both realistic and nominalistic terms. In particular, realistic interpretations assume that dominant species mutually restrict each other to discrete distribution areas and provide conditions to which certain other species adapt (Kirchhoff, 2007; Kirchhoff & Voigt, 2010). This results in delimited hierarchical systems of competition or food webs with a characteristic species composition. Nominalism arises with the refusal of such clear limitations and the assumption that the ecological relationships of every species have an individual reach; each species then has its ‘own’ association (Kirchhoff & Voigt, 2010). According to aggregationistic individualism, the ‘community’ or ‘association’ is a temporary juxtaposition of different species with more or less distinct spatial boundaries or continuous transitions delimited by *environmental conditions* as well as the *species’ specific requirements*. Other positions go even further: Neither the environmental conditions nor the plants themselves provide the observer with the criteria to subdivide vegetation into units. However, in order to orient oneself in the multitude of different combinations occurring in space and time, it may be useful for the ecologist to give names to some of these combinations according to his or her research interest. Therefore, the term ‘association’ has *heuristic usefulness* above all. Some have even refused to use the term ‘association’; for example Fritz Peus considered ‘association’ and ‘biocoenosis’ as “constructs of the human imagination” and did not accept them as objects of the natural sciences (Peus, 1954: 300, translated; cf. Kirchhoff & Voigt, 2010; Trepl & Voigt, 2011).

Thus, the analogies between liberal philosophies and individualistic theories in ecology consist primarily in the assumption of the (functional) independence of individuals from each other and from the supra-individual unit as well as the benefit orientation of their actions. Competition is the motor of development and the principle that structures society. In principle, development is unfinished.

### Analogies between democratic philosophies and synecological theories: the machine-like community

The progressive, rationalist theories of democracy of the French Enlightenment, and the many variants of socialism inspired by them, design society as a ‘machine-like community’. The idea that the world is determined by higher, reasonable and recognizable principles distinctively characterizes rationalism. In progressive rationalist political philosophies, the aim is to realize these rational principles in the construction of the state: therefore, the state is a comprehensive functional whole, a ‘machine’. Genevan philosopher Jean-Jacques Rousseau’s (1712–1778) philosophy is chosen here as the basis for the ideal type of democratism because Rousseau developed a political philosophy of ideal democracy. In contrast, French revolutionaries such as Robespierre or Saint-Just saw their task as one of transforming France into a democratic republic, that is, solving practical problems. They used theoretical arguments primarily for self-understanding or even for the justification of their actions after the fact (Fetscher, 1990: 258). In doing so, they referred largely to Rousseau, but ultimately not consistently, and thus produced contradictions within the theory. In addition, Rousseau’s philosophy is a theory of direct democracy without any liberal correction (ibid.: 144). Therefore, Rousseau—despite his special position among French Enlightenment thinkers, his pessimism about progress, and the multifarious ways in which he was interpreted—is well suited for the presentation of the political philosophy of democratism, since he developed it consistently. Analogies to this figure of thought can be found in early ecosystem theories from e.g. George E. Hutchinson (1903–1991) or Eugene P. Odum (1913–2002). In selecting the systems theories of Hutchinson and E. P. Odum, one criterion was the fact that they are considered influential with respect to the ecosystem approach (cf. Corman et al., 2019; Slack, 2010). Most importantly, I chose them because the differences between them can be shown very well through the discussion guided by my ideal types, so that statements can be made about the differences between at least two ecosystem concepts. Since both theories are considered classical, there is some evidence to suggest that these differences are ones that apply to a wide range of ecosystem theories.

(a) Following Rousseau’s philosophy, *individuals together construct the democratic state by subjecting their own will* to the general and common-good will (*volonté générale*) and thereby they become citizens. “Each of us collectively places his person and all his strength under the supreme direction of the general will, and we accept each member as an inseparable part of the whole” (Rousseau, 1762: I, VI, 44, translated). Individuals each have a ‘double character’, i.e. they are subject to government and the laws, and at the same time part of the sovereign, for they establish the government. “The people who are subject to the laws must also be their author; only those who unite are responsible for regulating the conditions of unification” (ibid.: II, VI, 71, translated). Since everyone participates with his free individual will in the formation of the general will and thus gives himself laws, he obeys when he follows the laws, “although he unites himself with all”, “nevertheless only himself” (ibid.: I, VI, 43, translated). The subordination of people to the general will results not only in the protection of the individual from exploitation by others, but also in the legal and social equality of citizens, insofar as everyone is equally, unconditionally and completely absorbed into society. The citizen has no individuality, which he or she has to develop organically as in conservatism. Everyone has the potential to fulfil any role and trains his or her abilities according to the interests of society, which ideally coincide with the individual’s own interests. This also means that citizens abstract from their natural differences and put aside their individual self-interest in order to establish the political body. Therefore, competition cannot arise nor can diversity develop in the community. The democratic society benefits the citizens by enabling the realization of *what is best for all of them.*

In the field of ecology, Hutchinson’s (1948) biogeochemical circulation system is a ‘machine-like community’. The system includes all organisms and abiotic elements involved in maintaining the nutrient cycle. All that constitutes the environment of the community in other theories is now treated as abiotic components of the system. As biotic components of the system, organisms are *dependent on the system* and at the same time, they *form it*. The essential properties of the components result from the functions they fulfil for the system, such as biomass production, transport or release of carbon compounds, etc. As parts of the same functional unit, organisms are *equivalent*, insofar as their participation in the system is equally necessary for maintaining the cycle of materials. Hutchinson does not conceive of organisms as competing with others or acting to meet their individual needs (or the needs of others)—they merely perform functions for the cycle of matter. In the same way, E. P. Odum’s ecosystem theory does not regard organisms in terms of the characteristics of their respective species. Organisms or groups of organisms that perform the same function in the ecosystem build ‘ecosystem compartments’ (Odum, 1971: 28). The biotic components are groups of functionally equivalent species, such as producers, macro- and micro-consumers (ibid.: 8). They are studied with regard to their contribution to energy conversion and storage processes etc. (rather than their species properties or relationships with other species). The components are functional for the ecosystem as a whole; they develop the ecosystem, bring about its dynamic stability and maintain it against disturbances (ibid.: 9).^5^ Ecosystem theories are *reductionist* in the (methodological) sense that the systemic perspective reduces the diversity of the properties of the ecosystem components to a very small number and considers them only with regard to a few, mostly physical parameters. In the process, the subject of the relationship between individual and community discussed in the organism-individualism debate, as well as the question of why species coexist, take a back seat (Voigt, 2011).

In democratic philosophy, the union of individuals into a community serves the purpose of *realizing the common virtues* of liberty, fraternity, and justice. These virtues guide all individual actions and they can only be realized in the community. According to ecosystem theory, the ecosystem is ‘constructed’ by its constituent components, not as an end in itself, but as a system of physical equilibrium. To what extent do organisms unite to serve a ‘purpose’? Ecological theories assume neither that organisms act in accordance with ‘higher purposes’, nor that the ecosystem as a whole has a purpose. However, one could argue that the fulfilment of the needs of each individual organism coincides with that of all others in a way that results in a system. In this way, the organisms in their actions ‘create the system’ and at the same time function to its benefit. This union is suitable for the fulfilment of purposes, even if there is no intention behind it. In the ecosystem perspective, organisms join together to form a community that maintains a self-regulating equilibrium through feedback, circulates substances, keeps itself from thermodynamic heat death by storing and utilizing energy, etc. The individuals of the natural world are not rational, nor do they know these general principles (only the ecologist who studies the synecological units do so), but are simply subject to them and thus ‘construct’ the community.

(b) In both democratic theory and ecosystem theories, a change in the community is a ‘constructional achievement’ of its components. These improve, for example, its efficiency with regard to the common realization of the general principles (and thus also with regard to the common will).

(c) In liberalism, the state and its legislation are useful instruments for limiting the negative effects of conflicting individual interests. In democratism, by contrast, the citizens are the ‘authors’ of the *contrat social* and the constructors of the state, but it is not their individual interests they express, but the common will. This will refers to principles that are a priori and *ahistorical* and have absolute universal validity—independent of the recognition and the realization of the principles of state order. Citizens have to realize *liberté, égalité*, and *fraternité* in their individual as well as their collective actions. They can recognize these principles through reason, which enables an insight into the general a priori order of the world—independent of every experience and every individual interest. Compared to conservatism, this reason does not enable the individuals to recognize and realize their particular position in the given hierarchical order. Rather, it enables the recognition of general principles that apply equally to all. Thus, the state is a realization of a priori principles through the citizens.

Concerning the ontological status of the ecosystem, positions differ. Ecosystems are natural units that are self-delimiting through their functional relationships and that genuinely exist to be discovered in nature (system realism). In this perspective they are constructed by their components in a mechanistic way. In other ecosystem theories, however, ecosystems are conceived as interest-dependent constructions of the scientist (system nominalism). The criteria for the selection of the system’s components and its delimitation are determined on the basis of a scientific interest or the particular observer’s interest in its use. Exactly those objects are components of the ecosystem that are relevant to this interest, e.g. ecosystem services such as production of biomass or stabilization of the climate. Since any number of interests are possible, an unlimited number of ecosystems can be defined. This means that an ecosystem is related to an externally defined purpose for which it is ‘mentally constructed’ (Voigt, 2009: 235ff, 2011; Kirchhoff & Voigt, 2010).

The analogies between democratic philosophies and certain ecosystem theories thus consist in the fact that individuals create the supra-individual unit neither by the relationships between individuals (as in the society of independent individuals), nor through manifold hierarchical dependencies (as in the organismic community), but through individuals’ acting in a way that is functionally related to the whole and its purpose. Individuals are both components and constructors of the whole. Therefore, they are dependent on and always related to the whole. The community realizes ‘general principles’ (and not an individual, diverse and hierarchical order).

### Analogies between conservative philosophies and synecological theories: ‘organismic communities’

Concepts and metaphors of ‘society as an organism’ have been widespread in political and sociological theories. They usually treat the relationships between parts of the community, i.e. individuals, in organic-functional terms. In contrast, the ideal type of conservatism emphasizes the individual’s specificity and unique character. Therefore, I construct the philosophy of early classical, Central European conservatism ideal-typically as envisaging an ‘organismic community’ and not an ‘organic community’. I substantiate this primarily on the basis of the cultural theory formulated by Johann Gottfried Herder in his “Ideas on the Philosophy of the History of Mankind” (1784-1791/1966) as well as excerpts from Adam Heinrich Müller’s (1809/2006) philosophy of the state. I have chosen these theories because, firstly, in them ‘organismic community’ is not an accidental metaphor, but part of the systematic and organizing structural core of conservatism, which emphasizes individuality. People develop by shaping themselves in an individual way according to their inner being and their outer bonds. Since people fulfil their tasks for and in the community in an individual way, they are not organs, but individuals. On the other hand, I have chosen these early conservative positions because they are firmly opposed to progressive liberal and democratic philosophies as well as to progressive social phenomena and early capitalism; later conservative positions, however, integrate both capitalism and democracy (Greiffenhagen, 1986).

In addition, certain ecological theories mostly referred to as organicist understand supra-individual units as ‘organismic communities’. This structural core becomes clear in early theories, e.g. those of Karl Friederichs (1878–1969) and August F. Thienemann (1882–1960).

(a) Early conservatism argues that people are *necessarily unequal* (Müller, 1809: 141, 216, 285). God, nature or tradition has given them different preconditions, roles and tasks. Thus, individuals interact with each other in a variety of individual and reciprocal ways. They have to take on their individual roles, e.g. practicing a profession, taking care of the family, being politically or culturally active etc. in accordance with tradition, family lineage, social status, etc. They fulfil tasks for others and therefore contribute to institutions such as family, feudal estate, and class, and thus to the community as a whole. In this way, individuals develop in a specific way according to their goals as determined by their inner nature and outer bonds. Doing so, individually and at the same time oriented towards the whole, they confirm the institutions of the state and bring them into being. In addition, freedom is implied by the realization of one’s own ties—and not, as in liberalism, by emancipation from precisely these ties. Since everyone should consider the whole in his actions, early conservatism vehemently criticizes the capitalist economic system and liberal theory, which are based on competition, isolation and egoism of individuals. The conservative community is not a society of equal, independent citizens, regulated by general laws, but a hierarchy characterized by diverse individual mutual relations.

This community is an end in itself to which the actions of each individual are subordinated and adapted. The state serves “all conceivable purposes because it serves itself” (ibid.: 68, translated).

In ecology, analogies come through very clearly in the writings of early German ecologist, August F. Thienemann. “Nature […], from the smallest spot in a meadow to the whole universe, is everywhere a closed living organism in which every smallest constituent is tuned to every other; every change in one part affects all others” (Thienemann, 1944: 35f, translated). “The community is […] a (supra-individual) wholeness, a coexistence and a mutuality of organisms” (Thienemann, 1939: 275, translated). The community is a functional and hierarchically organized whole and an end in itself. The individuals relate to another in such a way that they contribute to the formation of functional units. The units fulfil tasks which are different and of differing importance in and for the community: there are leadership functions and subordinate functions. At the same time, organisms depend on the community’s functioning—every ‘organ’ can only exist as a part of the whole community.

Accordingly, the analogy is that individuals are, usually obligatorily, functionally dependent on each other. They not only stand in dependent relationships with other individuals, but they also build functional groups (‘organs’ of the community). Individuals are thus ascribed different and differently important functions in and for the supra-individual whole, without which the latter could not exist; at the same time, each individual can only exist because of the functions of the others and the functioning of the community. Thus, individuals do not only act to meet their own particular needs; their needs correspond to a given function in the organ as well as in the holistic context.

(b) Historical development consists—as Johann Gottfried Herder has paradigmatically formulated (1784–1791)—in the individual and purposeful perfection of what is conceived as their individual ‘characters’ and what is laid out in their ‘living spaces’ by every people (‘*Volk*’). Development, therefore, ties in with what currently exists and respects what is ‘always valid’, for only in this way can continuity be maintained. The individual, sensitive adaptation of the people to the specific conditions of their habitat is at the same time a release from the immediate constraints of nature. This release is not an emancipatory cutting of specific ties, but an active, cultural and creative shaping of the landscape (Eisel, 1992). Individuality and diversity increases in the course of development.

Concerning succession, the organicist position postulates a *purposeful and goal-orientated development* from poorly integrated random pioneer societies to highly integrated organic and distinct units, characterized by an increase in reciprocal, obligatory relationships between organisms. During the course of succession, through its effects on the habitat the community produces its characteristic abiotic conditions (such as soil properties and microclimate) under which it can permanently exist. Succession proceeds to a final state, the climax, whereby certain environmental conditions (mainly macroclimatic) set unchangeable framework conditions. This climax is a ‘normal state’ in the sense not only that communities usually achieve it, but that they *should* achieve it (Trepl & Voigt, 2011). The attributes associated with the development, such as internal differentiation, functional integration and stability, are then both necessary attributes and a measure of the achieved level of development. Accordingly, organicist theories interpret deviations from the climax as undesirable developments. From the perspective of the community, the successional process of replacement of species means the *adaptation* of the whole to environmental conditions. The community adapts to the external conditions and shapes them; this implies a liberation from direct natural constraints. The community’s development is purposeful: it realizes what is ‘intended’ as a potential in the community and in its external conditions.

(c) According to conservatism, there is no difference between the people and the state. Man needs the state to be man and “is not to be thought of outside the state” (Müller, 1809: 40, translated). The state is a social order that has arisen and developed by itself. Living in a community is ‘natural’; it is not an arbitrary decision made by individual persons, but is a priori to the individual (e.g. ibid.: 62). The state and its institutions do not owe themselves to a contract out of the will of autonomous citizens, but are ‘realistic’, natural and historically grown.

Organismic communities are realistic units, which exist independently of the scientist; he or she cannot delimit them at his/her convenience, but must find these units and its boundaries in nature. They delimit themselves, because the organisms—either on their own or as parts of organs—are in obligatory dependency relationships with each other, and these relationships are constitutive for the wholeness of the community. Given that the invariable regional climate (as an external condition) and the species present in neighbouring areas (as an internal condition) specify which (climax) community will emerge, one could say that the community is already there ‘from the beginning’. The regional climate and the existing organisms contain the possibility of its emergence, which then only has to come into effect (cf. Kirchhoff & Voigt, 2010; Trepl & Voigt, 2011; see in detail Kirchhoff, 2007: 80 ff.; Voigt, 2009: 157 ff.). Early German representatives of organicism, such as Karl Friederichs and August Thienemann, countered scientific standards with an ‘intuitive’ and ‘contemplative view on nature’, which enables the researcher to identify these communities (Friederichs, 1957: 120; Thienemann, 1954: 322, 317).

The analogies between conservative philosophies and organicist theories thus consist primarily in the fact that individuals are in (mostly obligatory) dependencies to each other, to organs of the community and to the community as a whole. Individuals fulfil tasks in the diverse, functional and developmental context of the organismic community. The community precedes the individual; it produces itself as a whole. Its development is continuous and goal-oriented, it realizes what is ‘laid out’ as a possibility in the community and in its external conditions, and in doing so it detaches itself from direct natural constraints. The supra-individual unit (people and state as well as community) is natural and realistic.

## Heuristic benefits of analogies

What have we learned from discussing ecological theories against the backdrop of the ideal types of political positions and the identification of the above parallels? In the discussion that follows, I focus on the benefit that the precise formulation of assumptions about ‘individuality’ and ‘action-guiding purposes’ in different political philosophies can bring for the philosophy of ecology.

### Heuristic benefits for the philosophy of ecology: assumptions about ‘individuality’ and ‘action-guiding purposes’

On the basis of analogies with political philosophies, one can distinguish and discuss ecological theories from the point of view of the philosophy of science. Of course, this is also possible with the help of other methods, but the one chosen here has some advantages. Analogies to political philosophies open up completely different possibilities for distinguishing between ecological theories than the conventional classification into reductionistic and holistic, which is based above all on the relationship between parts and wholes. In contrast, political philosophies differ because of divergent ideas about the essential characteristics of the *individual* as well as about *purposes*: Is he or she an independent egoist, an individual but dependent part of a community, or a citizen showing solidarity with the rest of society? Do the actions of individuals benefit or harm only themselves or do they fulfil functions for others and/or the whole? Does the whole benefit the individual, is it an end in itself or does it benefit the citizens by enabling the realization of what is best for all of them? Assumptions about ‘individuality’ and ‘action-guiding purposes’ are decisive for political philosophies and therefore *precisely* formulated in defence against political opponents. How do such assumptions about individuality and purpose fare in biology, the science that studies living organisms? On one hand, living nature is often spoken of in a way that seems to be diametrically opposed to the scientific perspective in which everything appears to be a value-free object of theoretical knowledge (Spaemann & Löw, 2005; Trepl, 2005), because the concept of the living organism seems to imply that ‘self-maintenance’ and ‘developmental goals’ can be meaningfully applied. When used in relation to the organism, these terms assume that the state of life is the purpose and desirable goal of the organism. On the other hand, in science it is not possible to assert that things happen in nature according to some purpose. Orientation to purposes presupposes the idea of a purpose preceding a cause, that is, an action based on an intention; but we cannot suppose that nature has intentions. If we do, we are not considering it scientifically. After all, it is the fundamental assumption of ecology that organisms are individuals that strive to maintain and reproduce themselves and therefore ‘behave in a purposeful way’—but this assumption has only a heuristic status. Ecology can neither ignore nor explicitly address or define and discuss this fundamental assumption as such (Toepfer, 2004). Thus, the benefit of discussing ecological theories in terms of their analogies to political philosophies consists in the ability to identify differences and peculiarities of the ways *individuality* and *intentional action* are approached in different political philosophies—something that is not possible in this way in ecology as a natural science. I illustrate the heuristic utility of this approach with three examples.

(a) In the history of ecology, the theory of Frederic E. Clements (1874–1945) is usually presented as the standard example of organicism, characterized by the assumption that communities are organized and develop like an organism, i.e. that they are functionally integrated units of mutually dependent species. As has been quoted many times, he called the vegetational formation “a complex organism, or superorganism” (Clements & Shelford, 1939: 20). “The unit of vegetation, the climax formation, is an organic entity. As an organism, the formation arises, grows, matures and dies” (Clements, 1936: 261). However, if we discuss Clements’ theory against the background of the ideal–typical organismic community, then it becomes clear that it differs fundamentally from both the conservative theory of an organismic community and the usual conception of the organization of an individual organism. It turns out that plant communities are to a large extent the result of competition for resources (e.g. Clements 1916: 72; Clements & Shelford, 1939: 162 f.),^6^ as it corresponds to the liberal view of interactions, and not as an outcome of manifold mutual dependencies between a diversity of species. However, in order not to contradict the logical structure of the ‘organismic community’, he assigns competition a different role than it is given in liberalism, seeing it as functional for the development and hierarchical organization of the community. One could say that Clements’ view proceeds in a similar way to ‘modernized’ conservatism; conservatism had initially rejected capitalist competition (or its liberal interpretation), but later integrated it into the concept of the organismic community, interpreting it conservatively (Voigt, 2009: 88 ff.).^7^ This example shows that focusing on questions about the causes and factors of the coexistence of individuals/species as well as about the nature of the relations between individuals/species can reveal new aspects to a theory that is considered paradigmatic for organicism, but contradicts it in respect of the nature of the determinant interactions. The comparison with the three ideal–typical answers to these questions from the area of political philosophy makes it possible to identify this discrepancy.

(b) Another example is Gleason’s individualistic theory, usually presented as the standard example of individualism. Indeed, Gleason’s theory corresponds relatively well with the “society of independent individuals” as formulated in early liberalism: the existence of an organism in a society is determined by the fact that its requirements are met there, but beyond that, organisms are independent individuals. However, in contrast to the ideal type, for Gleason neither competition for resources nor the displacement of organisms by others that are more competitive is decisive for the structure of the association or its succession. “The location and behavior of every individual plant is determined partly by heritable requirements and partly by the environmental complex under which it grows” (Gleason, 1927: 321); “vegetation is the resultant of migration and environmental selection” (Gleason 1926: 24). It remains open whether he assumes that there are only a very small number of interactions, or whether they take place but do not have a pivotal impact. However, Gleason does not deny that the environment of a plant species also includes other species (ibid.: 10), nor that species can correlate in their distribution (Gleason, 1939: 108). The influence of other species acts only indirectly by affecting abiotic site conditions (Gleason, 1926: 17), with exceptions such as parasites. Even if interactions do not explain the coexistence of species in an association, their composition is not arbitrary, but determined by environmental selection. In contrast to interactionist ecologists as Ramensky, Gleason holds an aggregationistic individualist position (Kirchhoff & Voigt, 2010).^8^ ‘Community’ or ‘association’ is merely a name for a group of individuals gathered together more or less at random at a given moment in a place where they find suitable environmental conditions. It can be said that the comparison of Gleason’s theory with the ideal type of liberalism makes obvious their differences concerning the role of interaction between individuals in the formation of society. In this example, too, the method facilitates the identification of specific features of a given theory. In addition, it makes it possible to distinguish aggregationist from interactionist theories in ecology.

(c) Moreover, the method allows us to differentiate between two different types of theories about functional wholes. With a focus on *purposes* and *individuality*, one can distinguish *organismic* from *machine-like wholes*. In the former, the individual is involved in manifold mutual relationships with other individuals; it is part of various functional units and its actions relate to the whole. This whole is a community that individually differentiates itself. In contrast, in machine-like communities, the individuals are nothing more than holders of functions in and for a whole. This whole is organized for accomplishing certain principles, but it is neither individual nor diverse. In this case, too, the distinction between different purposes and concepts of individuality in political philosophies makes it easier to differentiate between ecological theories. However, as the prominent example of Eugene P. Odum’s (1913–2002) theory shows, the ecosystem approach also makes it possible to reformulate organicism. Following Odum (1971), an ecosystem consists of abiotic and biotic components. In accordance with the machine-like community, the biotic components are groups of species (e.g., producers, consumers and decomposers); each group consists of species that are equivalent in terms of functionality. Dependencies bind them together, but above all, species fulfil functions for the ecosystem as a whole, insofar as they develop it to the climax state and preserve it against disturbances. In contrast, the main organicist aspect is that Odum, while emphasizing the functional equivalence of species, emphasizes obligatory interspecific relationships, among them relationships of mutual dependence. For Odum, the ecosystem approach allows one “to emphasize obligatory relationships, interdependence, and causal relationships, that is, the coupling of components to form functional units” (Odum, 1971: 9). As the number of these relationships increases and competition decreases in succession, the goal-oriented development leads to a stable, diverse climatic ecosystem. The climax species work together to perpetuate the nutrient cycle, to use energy efficiently and thus preserve the ecosystem. Consequently, ecosystems are systems demarcated due to their components’ interspecific relations and functional dependencies. Odum reduces the species to functions and the functions to contributions to material cycles and energy flows in an ecosystem-based manner; therefore, his organism is a *reductionist organism* (Voigt, 2009: 209 ff.; Kirchhoff & Voigt, 2010). Thus, Odum’s theory of ecosystems shows significant analogies both to aspects of the ideal type of the ‘organismic community’ and to the ‘machine-like community’.

These three examples show that by incorporating the notion of analogical pairings between political philosophy and ecological theory, students of the latter can be made more aware of the distinctive characteristics both of those theories which are considered ‘typical’ of particular viewpoints and of those which are more divergent.

### Heuristic benefits for the science of ecology

The method of analysing and discussing ecological theories using analogies to political philosophies is not only relevant to the theory and history of science. Even empirically working ecologists follow a theory. The exploration of its political analogue broadens the view and enables one to expand one’s own argumentation, to consider alternatives or prevent self-contradiction, to forestall criticism and to recognize the weak points of opposing theories.

With Luhmann (1997) one could say that this method enables the ecologist to assume *a second-order observer position*. Luhmann defined “observation” as the general operation of drawing a distinction and making an identification. The second-order observer knows that the first-order observer (who may be the very same person) creates the world through a distinction invisible to him/her. Moreover, second-order observers know that it is only by means of this specific distinction and designation that the first-order observer gets the opportunity to see his or her ‘own’ object—e.g. organismic communities, ecosystem machines, competitive relationships or a juxtaposition of immigrant species with similar requirements. Second-order observers observe *which distinctions* the first-order observer makes use of in order to observe and thus they recognize the ‘blind spots’ which cannot be perceived in the first-order observation. To the extent that second-order observers are able to observe *how* they observe or make distinctions, they always gain knowledge about themselves and their scientific paradigms. Therefore, second-order observers can extend their own first-order observation to include alternatives, because now they not only see what they see, but also see that there are things they do not see, but might have seen if they had adopted a different perspective. They know that there exist other possible ways to look at the world which they could adopt instead. Therefore, it makes sense even for ecologists working empirically or in object theory to ask themselves about the ‘how’ of their observations and to consider alternatives.

It may even be heuristically fruitful to get inspiration from analogical counterparts for the elaboration of theories of ecology, e.g. for the development of ecological competition theories from current economic competition theories. Of course, it will not be possible to formulate and empirically verify everything contained in the latter as a consistent theory in the ecological domain. However, for the formulation of synecological theories one can receive inspiration from the theories of supra-individual units of completely different scientific disciplines or areas of life. The method is therefore useful for the *context of discovery*, not for the context of justification.

The method also allows one to recognize (and I can only hint at this in the context of this article) the fundamentally political character of the goals of nature conservation, which are often formulated not as societal goals but as ecological facts or requirements. Ecological theories are often explicitly or implicitly used as a basis for societal decisions in nature conservation. Their selection includes or excludes certain patterns of action. For example, the concept of a ‘closed’ organismic community suggests a different way of dealing with biological invasions than the concept of an ‘open’ society of independent individuals, since the consequences of invasions for the synecological unit are different depending on the theory.

## The constitution-theoretical hypothesis: the ecological discourse is structured by modern paradigms of socialization

The observation of analogies leads to the question of the relationship between scientific theory and political conceptions of human society. The existence of valuable analogies does not mean that the same processes or dependencies apply in the realm of nature as in the realm of human society. Looked at from the perspective of a*theory of constitution*, social relations exist, which generate different conceptions of nature as ‘like this’ or ‘like that’ (Eisel, 2002).^9^ The theory of constitution is based on the scientific-theoretical criticism of empiricism and positivism, which has shown that science does *not* come to its conclusions—which are considered ahistorical and universal—by inductive generalization of specific empirical observations that are independent of theory and subject matter. Observations are always guided by theory; they cannot be ‘unprejudiced’. Scientific theories owe their existence to non-scientific conceptual structures already in existence. These ‘prejudices’ are a historical a priori (Foucault, 1972: 127 f.)^10^ or “cultural patterns of interpretation that are already available” (Eisel, 2002: 130, translated), which are the preconditions for the possibility of scientific concepts and their corresponding objective experiences. Indeed, these conditions ensure that new facts do not destroy the old conceptions in general but consistently confirm the theory (or the paradigm) (Eisel, 2002; cf. Kuhn, 1962). These cultural patterns structure and constitute our thinking—not only scientific thinking, but our thinking in general. Constitutional ideas are, for example, competing images of man, ideas about ideal human coexistence and the corresponding political philosophies. They have arisen against the background of certain constellations of social problems in ways which are historically and culturally bound. The respective constellation of the social horizon of experience makes certain ideas possible while excluding others (Eisel, 2002).

Therefore, one important reason why different and competing positions exist in ecology can be recognized in the existence of social relations, which generate different and competing cultural ideas and ideals about the individual and society such as the political philosophies of liberalism, conservatism and democracy. In the empirical natural sciences, these cultural ideas are set up against one another and are differentiated as scientific theories. Scientific ecological theories arise from socially created images and expectations and competing cultural-historical ideas of human societies, or are at least inspired by them. Ecological paradigms can thus be understood as political ideas read back into the workings of nature (Trepl, 1994a, 1994b; Eisel, 2002: 130 ff.; Voigt, 2009: 54 ff.). Thus, to talk about nature is also to talk about (our expectations of) society, societal-cultural values, and the longings and fears people project onto nature. Moreover, these ecological theories, and the management goals and activities derived from them, can have the power to legitimize these socially created images and political expectations about society by means of scientific confirmation.

Therefore, an acknowledgement of the political character of scientific theories enables a profound critique of naturalistic views (Eisel, 2004; Trepl, 1994a). It shows the implicit socio-cultural patterns of interpretation also in those scientific theories that underlie the naturalizing view of man and society within the social sciences. This can be shown with a concise example^11^: Following the theory of constitution presented above, Darwinian theory in biology is based on certain ideas of constitution. These ideas are based on the experience of the economic and social form of organization in ‘Manchester capitalism’ and the cultural and political processing of this experience within a specific worldview (liberalism). These socio-cultural constitutional ideas thus have political content. They exist as intersubjective patterns of thought for the interpretation of nature and can be ‘read into’ nature or formulated as a scientific theory (Darwinism). Darwin ‘saw’ the social conditions of early capitalism in the view of liberalism’s conception of man and society and ‘found’ them in the modes of nature. As a result, the modes of nature could now be used ‘naively’ and seemingly independent of social interests for the explanation and legitimation of social conditions or the legitimation of political ideologies (Social Darwinism). A circle is created. Its first part, the cultural constitution of scientific objects and theories, cannot and should not be avoided; it can only be acknowledged and taken into account, because it is an inevitable theoretical aspect of scientific heuristics. But the second part, the process of retransmission and the concealment of political interests with reference to the way nature ‘exists’, can certainly be criticized (Eisel, 2004: 29, 38–42).

## Conclusion

I have tried to show that comparison between classical political philosophies and classical ecological theories represents a fruitful approach, enabling us to uncover new aspects of well-known historical theories of ecology. In each case, I have referred to a core that is easily recognizable both in early political philosophies and in early ecological theories. However, political philosophies do not remain bound to their original historical interests, but are transformed and adapted to new social conditions. The same applies to ecological theories, which must take into account the arguments of competing theories as well as empirical facts, and differentiate themselves. To hint briefly at how to situate more recent theories against the backdrop of analogies to political theories, I give just a few examples from the broad spectrum of theory development in ecology.

Various theories show analogies to this ideal type of the ‘society of independent individuals’– theories that can even be in direct conflict from a different point of view, especially as regards interspecific relations and the role attributed to them in the coexistence of species. In the 1950s and 1960s, aggregationistic continuum theories (e.g., Curtis & McIntosh, 1951; McIntosh, 1967; Whittaker, 1953, 1956) were influential. They emphasize that discrete associations delimited by themselves do not exist in nature. Species composition changes more or less continuously because the suitability of a site varies continuously, each species has individual site requirements, and a species is not tied to particular other species (Kirchhoff & Voigt, 2010). Subsequently, interactionist equilibrium theories came to the fore, which particularly emphasized the relevance of competition (niche theories, e.g., Hutchinson, 1957, 1959; Lack, 1944; MacArthur, 1958, 1972). The competitive relations between species lead to coevolutionary niche differentiation and, in succession, repeatedly to certain equilibrium states in which the many niches are divided among the most competitive species (Kirchhoff & Voigt, 2010: 187). These theories show analogies to the ‘society of independent individuals’ in that individual species do not perform functions for a community. Species can coexist not because of but in spite of competition. Consequently, disequilibrium theories (e.g., Connell, 1978; Pickett, 1980) emphasized that there is no balance of species composition when, due to fluctuating environmental conditions, the relative competitiveness of different species changes sufficiently rapidly. Other positions criticized these interactionist theories, which assume equilibrium, from the position of aggregationism. They did not agree that competition for resources has a major impact on the distribution of species, as population densities often lie below the capacity limits of the resources involved (e.g. Wiens, 1984). Are there more recent positions in which analogies to the organismic community can be found? One finds such positions mainly in popular scientific writing and also in large parts of the environmental movement. In modified form, however, the structural core has continued to be promoted in ecology, namely in the form of ecosystem theories, as I have shown using Odum as an example. But other ecosystem theories are explicitly driven by technical interests. These do not regard ecosystems as natural entities, but as artificial entities. The criteria for the selection of components and the delimitation of ecosystems are determined on the basis of an interest in use: Exactly those objects are components of the ecosystem that are relevant for the respective interest, e.g. for the production of biomass or for the stabilization of the climate (ecosystem services).

It would be an interesting task to search for current, perhaps radically different theories on the relationship between state, individual and society and to examine to what extent these can be used heuristically to discuss today’s theories of ecology.

However, as far as the history of earlier theories of ecology is concerned, the political reading of ecological theories can shed light on controversial debates in ecology, and also on the associated planning practices and ideas of value—for example as concerns dealing with biological invasions in nature conservation. Scientific theories can be designated as political insofar as they are constructions of social self-legitimation projected into nature. Furthermore, the theory of constitution shows that debates about scientific theories contain more than the question whether theories of a certain type describe certain natural phenomena correctly: there is also the question of the conflict between ideas about the ‘correct’ relationship between individual and community.

## Footnotes

1 For the debate on the holism-reductionism controversy in ecology, see e.g. the contributions in Saarinen (1982), Schwarz and Jax (2011), McIntosh (1985), Hagen (1992), Golley (1993), Worster (1994), Bergandi and Blandin (1998), Keller and Golley (2000), Eliot (2011). See also the overview in Voigt (2012).

2 Differences between ecological theories and structurally analogous political philosophies result, for example, from the fact that in the latter, but not in the former, it is assumed that the individual is endowed with reason and thus that he or she has the freedom to behave ‘wrongly’, i.e. not according to the social model striven for as an ideal. I will not go into these differences further, since I am interested in showing not the differences but the structural similarities between these two fields.

3 In addition, structural similarities between political theories and other theories of biology have often been pointed out, see e.g. Nordenskiöld (1926: 464–467, 484 ff.), Mitman (1988), Worster (1994), Depew and Weber (1995: 7, 122 f.), Gould (1999: 92 f.).

4 I refer hereby to earlier work (Voigt 2009) and build on it.

5 For a further discussion of Odum’s theory, see Sect. 4.1.

6 Following Clements, in succession individuals of a layered forest compete with each other at first in so far as they are part of the same layer. “With the growth of shrubs, the latter become dominant over the herbs and are in turn dominated by the trees. Herbs still compete with herbs, and shrubs with shrubs, as well as with younger individuals of the next higher layer. Within the dominant tree-layer, individuals compete with individuals and species with species. Each layer exemplifies the rule that plants similar in demands compete when in the same area, while those with dissimilar demands show the relation of dominance and subordination” (Clements 1916: 72).

7 See Kirchhoff (2007) as well as Kirchhoff (2020), this volume, for another interpretation of Clements’ concept. Kirchhoff examines the aspect of determination by so-called dominants and explains Clements’ concept of the plant community as a top-down control-hierarchical entity.

8 See in detail Kirchhoff (2007: 187 ff.), Voigt (2009: 167 ff). In contrast, Nicolson and McIntosh (2002) argue that Gleason’s concept has commonly been ‘misconstrued’ as asserting that the community is a random collection of species, and the species are responding solely to the abiotic environment. They emphasize that Gleason accorded crucial importance to interactions between and among organisms.

9 In this context, the meaning of constitution is different from the standard sense in philosophy, as seen in the work of e.g. Immanuel Kant (1724-1804). 

10 By ‘historical a priori’ Foucault refers to the fact that the conditions that make thinking possible are necessary as well as historically contingent. They are necessary in the sense that we cannot think without or outside of these conditions, and contingent in the sense that they have been different before, and could be again in future.

11 See in detail and with different perspectives Nordenskiöld (1926: 464–467, 484 ff.); Depew and Weber (1995: 7, 122 f.), and Gould (1999: 92 f.).
